# Supplementary material for: The Rate of Avoidable Pancreatic Resections at a High-Volume Center: An Internal Quality Control and Critical Review
Source: J Clin Med. 2023 Feb 17;12(4):1625. doi: 10.3390/jcm12041625 (PMC9967180; doi:10.3390/jcm12041625)
Supplement: Supplementary file 1 [file jcm-12-01625-s001.zip › jcm-2182393-supplementary.pdf]

Supplementary Table S1. Mismatches: critical review of the diagnostic workup

Patient no.	Year	Symptoms	Clinical suspicion	Histologic diagnosis	Tissue sampling	Histology tissue sampling	Preoperative workup	Radiological aspect	Missing preop. investigations and/or pitfalls and/or mistakes	Mismatch
**#1**	2003	none	PDAC	Intrapancreatic accessory spleen	EUS-FNA	N/C	-Abdomen CT<br>-MR<br>-EUS+FNA<br>-TM: Ca 19.9 65 U/mL, CEA neg.	-MR: suspected PDAC<br>-CT: no lesion<br>-EUS: suspected PDAC	-No Tumorboard discussion<br>-No second sampling attempt (preferably FNB)<br>-No tri-phase CT (pancreas protocol)<br>-No ^{99m} Tc-HDRBC scintigraphy-SPECT/CT	MAJOR
**#2**	2009	none	pNET vs CP	No solid lesion, PanIN 1	not performed	-	-PET-CT<br>-tri-phase CT<br>-TM: CgA 71.7 nmol/L	-PET-CT: pNET vs CP<br>-CT: hypervascular lesion (susp. pNET)	-No Tumorboard discussion<br>-No sampling attempts<br>-NSA not measured	MAJOR
**#3**	2010	Abdominal pain	PDAC	AP	EUS-FNB	N/C	-Abdomen CT<br>-ERCP<br>-EUS-FNB	-CT: pancreatic lesion not clearly recognized<br>-ERCP: stenosis of pancreatic duct<br>-EUS: report not available	-No Tumorboard discussion<br>-No second sampling attempt<br>-TM not measured<br>-IgG 4 not measured<br>-No tri-phase CT	MAJOR
**#4**	2010	Obstructive jaundice	Vater's papilla adenocarcinoma vs PDAC	Aspecific flogosis Vater's papilla	ERCP-FNB	N/C	-Abdomen CT<br>-ERCP	-Abdomen CT: double duct sign, lesion not recognizable<br>-ERCP: no papillary lesions recognizable	-No Tumorboard discussion<br>-No second sampling attempt<br>-TM not measured<br>-No tri-phase CT (pancreas protocol)<br>-additional EUS	MAJOR
**#5**	2012	Obstructive jaundice	Vater's papilla adenocarcinoma vs PDAC	Adenomyomatous hyperplasia Vater's papilla	not performed	-	-Abdomen CT<br>-ERCP+stent (2x)	-CT: no lesion recognizable<br>ERCP: prepapillary CBD stenosis	-No Tumorboard discussion<br>-No sampling attempts<br>-No tri-phase CT (pancreas protocol)<br>-TM not measured	MINOR
**#6**	2013	Obstructive jaundice	PDAC	AP	not performed	-	-MR<br>-ERCP+stent (3x)<br>-EUS<br>-TM: Ca 19.9 5351 U/mL, CEA neg.	-MR: dilated CDB<br>-EUS: hypovascular lesion uncinate process	-No Tumorboard discussion<br>-No sampling attempts<br>-IgG 4 not tested<br>-No tri-phase CT	MAJOR
**#7**	2013	none	pNET	Pancreatic lymphoepithelial cysts	EUS-FNA	N/C	-Abdomen CT<br>-EUS<br>-TM: Ca 19.9 397 U/mL, CEA neg.<br>-Tumor board	- CT: unclear lesion pancreas tail (solyd-cystic tumor)<br>-EUS: report not available	-No second sampling attempt<br>-No tri-phase CT (pancreas protocol)	MAJOR

PDAC= pancreatic ductal adenocarcinoma; pNET= pancreatic neuroendocrine tumor; AP= autoimmune pancreatitis; CP= chronic pancreatitis; EUS=endosonography; EGDS= esophago-gastro-duodenoscopy; ERCP= endoscopic retrograde colangio-pancreatography: CEA= carcino-embrionary antigen; Ca 19.9= carboidratic antigen 19.9; NSE= neuron specific enolase; CgA= cromogranin A; FNA=fine needle aspiration; FNB=fine needle biopsy; N/C= not conclusive; TM= tumor markers; CT= computed tomography; MR= magnetic resonance; LGD= low grade dysplasia; D/D= differential diagnosis; LGD= low grade dysplasia; IgG 4= immunoglobulin G 4; PanIN= pancreatic intraepithelial neoplasia; ^{99m} Tc-HDRBC scintigraphy-SPECT/CT= 9mTc-labelled heat-denaturated red blood cell scintigraphy-single-photon emission computed tomography.

Supplementary Table S2. Mismatches: critical review of the diagnostic workup										
**Patient no.**	**Year**	**Symptoms**	**Clinical suspicion**	**Histologic diagnosis**	**Tissue sampling**	**Histology tissue sampling**	**Preoperative workup**	**Radiological aspect**	**Missing preop. investigations and/or pitfalls and/or mistakes**	**Mismatch**
**#8**	2013	Resistant hypoglycemia	pNET	Pancreatic nesidioblastosis	not performed	-	-tri-phase CT<br>-Lab: C-Peptide, Insulin, Fasting test	-CT: hypervascular pancreatic lesion, suspected insulinoma	-No Tumorboard discussion<br>-No sampling attempts<br>-TM not measured	MINOR
**#9**	2014	Obstructive jaundice, history of CP	PDAC	AP	not performed	-	-tri-phase CT<br>-TM: Ca 19.9 neg<br>-Tumor board	-CT: suspected locally advanced PDAC (hypovascular solid lesion)	-No sampling attempts<br>-IgG 4 not measured	MAJOR
**#10**	2015	Obstructive jaundice	PDAC	Adenomyomatous hyperplasia Vater's papilla	not performed	-	-tri-phase CT<br>- TM: Ca 19.9 and CEA neg.<br>-Tumor board	-CT: CBD dilation, no lesion recognizable	- No sampling attempts<br>- additional imaging (if CT unclear): ERCP, EUS, MR	MINOR
**#11**	2015	Obstructive jaundice	PDAC	Adenomyoma of the common bile duct (HGD)	not performed	-	-tri-phase CT	-CT: suspected PDAC (hypovascular solid lesion)	-No Tumorboard discussion<br>-No sampling attempts<br>-TM not measured	MINOR
**#12**	2016	none	pNET	Intrapancreatic accessory spleen	not performed	-	-tri-phase CT<br>- TM: Ca 19.9 and CEA neg.<br>- ^{99m} Tc-HDRBC scintigraphy-SPECT/CT: negative	-CT: hypervascular pancreatic lesion	-No Tumorboard discussion<br>-No sampling attempts<br>-CgA and NSE not measured	MAJOR
**#13**	2016	Obstructive jaundice	PDAC	AP	not performed	-	-tri-phase CT<br>-ERCP+stent<br>-TM: Ca 19.9 38 U/mL, CEA neg.	-CT: suspected PDAC (hypovascular solid lesion)<br>-ERCP: stenotic CBD	-No Tumorboard discussion<br>-No sampling attempts<br>-IgG 4 not measured	MAJOR

PDAC= pancreatic ductal adenocarcinoma; pNET= pancreatic neuroendocrine tumor; AP= autoimmune pancreatitis; CP= chronic pancreatitis; EUS=endosonography; EGDS= esophago-gastro-duodenoscopy; ERCP= endoscopic retrograde colangio-pancreatography: CEA= carcino-embrionary antigen; Ca 19.9= carboidratic antigen 19.9; NSE= neuron specific enolase; CgA= cromogranin A; FNA=fine needle aspiration; FNB=fine needle biopsy; N/C= not conclusive; TM= tumor markers; CT= computed tomography; MR= magnetic resonance; LGD= low grade dysplasia; D/D= differential diagnosis; LGD= low grade dysplasia; IgG 4= immunoglobulin G 4; PanIN= pancreatic intraepithelial neoplasia; ^{99m} Tc-HDRBC scintigraphy-SPECT/CT= 9mTc-labelled heat-denaturated red blood cell scintigraphy-single-photon emission computed tomography.
